# Obstetric Perineal Tears, Birth Characteristics and the Association with Urinary Incontinence Among Primiparous Women 12 Months Postpartum: A Prospective Cohort Study

**DOI:** 10.1007/s00192-024-05920-2

**Published:** 2024-09-16

**Authors:** Ditte Gommesen, Sarah Hjorth, Ellen A. Nohr, Niels Qvist, Vibeke Rasch

**Affiliations:** Department of Gynecology and Obstetrics, Odense University Hospital, J.B. Winsløws Vej 4, 5000 Odense C, Denmark

**Keywords:** Obstetric perineal tears, Urinary incontinence, Female health, Post partum health

## Abstract

**Introduction and Hypothesis:**

Postpartum urinary incontinence (UI) is common and a concern for many women, as UI leads to a lower quality of life and self-esteem. Perineal tears may be a risk factor for UI, yet few studies have investigated the association between the degree of perineal tear and risk of developing UI postpartum. The objective was to examine how the degree of perineal tear and selected obstetric risk factors were associated with any UI and stress ultrasound (SUI) 12 months postpartum among primiparous women.

**Methods:**

A prospective cohort study was conducted at four Danish hospitals. Baseline data were obtained at a clinical examination 2 weeks postpartum. Symptoms of UI were evaluated 12 months postpartum by the International Consultation on Incontinence Questionnaire-Urinary Incontinence Short Form questionnaire (web-based). Multivariate regression analyses were performed to investigate the risk factors for UI.

**Results:**

A total of 603 primiparous women (203 with none/labia/first-degree tears, 200 with second-degree tears and 200 with third-/fourth-degree tears) were included between July 2015 and January 2018. Women with tears involving the perineal muscles reported any UI more often than women with no/labia or first-degree tears (spontaneous second-degree tear: RR 2.04, 95% CI 0.92–4.50; episiotomy: RR 2.22, 95% CI 0.99–4.96; third- or fourth-degree tear: RR 2.73, 95% CI 1.18–6.28). The same was found for SUI, but with wider confidence intervals.

**Conclusions:**

A higher prevalence of any UI and SUI was found among women with perineal tears involving any perineal muscles, compared with women with no, labia, or first-degree tears.

## Introduction

Urinary incontinence (UI) after vaginal delivery is a major concern for many women as UI leads to lower quality of life and self-esteem, with implications for social, mental, and physical health, and significant societal costs [1]. UI is defined as the complaint of involuntary loss of urine experienced during the bladder storage phase [2] and is a common complaint, with one third of all women reporting leakage that impacts their social or hygienic well-being [3]. It is divided into several subtypes according to the situations in which the leakage occurs, with stress UI (SUI) being the most common type [4]. SUI is defined as the complaint of involuntary loss of urine on effort or physical exertion (e.g., sporting activities), or on sneezing or coughing [2]. It is common after vaginal delivery, and a Canadian survey found that 23% of women had SUI 4 months postpartum [5]. However, the reported prevalence of UI postpartum varies markedly in the literature, from 3 to 40% [6, 7].

Both UI in general and SUI have been linked to increased age and body mass index (BMI) [8, 9]. However, most research on obstetric risk factors related to UI has focused on the mode of delivery, including caesarean section, operative vaginal delivery, and spontaneous vaginal delivery [10, 11]. Handa et al. found that women with perineal lacerations did not have an increased risk of SUI 5–10 years postpartum, whereas Borello-France et al. found no association between anal sphincter tears and developing UI compared with no anal sphincter tears or caesarean section [12, 13]. However, there is a lack of studies on how the degree of perineal tear and other obstetric factors are associated with the risk of developing UI postpartum. Thus, the objective of this study was to examine how the degree of perineal tear and selected obstetric risk factors were associated with any UI and SUI 12 months postpartum among primiparous women.

## Materials and Methods

### Study Setting

This study was part of a prospective cohort study conducted from July 2015 until January 2019 at four hospitals in Denmark: two university hospitals (Odense (OUH)) and Aarhus (AUH)), and two general hospitals (Esbjerg and Kolding). The study population was sampled in three groups stratified according to the degree of tear. A total of 603 primiparous women were included and sampled into three groups: 203 with none/labia/first-degree, 200 with second-degree and 200 with third-/fourth-degree tears. The sample size calculation is described in detail elsewhere [14].

### Inclusion and Follow-Up Procedure

Primiparous women, 18 years or older, able to read and speak Danish, were eligible. Exclusion criteria included stillbirth or critically ill newborns. Eligible women were informed about the study immediately after delivery and before discharge from the hospital. Interested women received further information via email and were contacted by phone and invited to participate in the study. Participants completed a baseline questionnaire, including the Danish version of the International Consultation on Incontinence Questionnaire-Urinary Incontinence Short Form (ICIQ-UI-SF) [15] within the first 21 days postpartum, providing information about pre-pregnancy UI. This was followed by a clinical examination (perineal inspection and digital palpation) to assess wound infection and healing, and to validate the degree of tear diagnosed at the delivery. At 12 months postpartum, participants received the ICIQ-UI-SF by email and were invited to a gynaecological examination followed by endovaginal ultrasound (EVUS) and endoanal ultrasound (EAUS) and three-dimensional high-resolution anorectal manometry (3D HRAM). The first author (DG) performed all EVUS, EAUS and 3D HRAM examinations. For EVUS and EAUS, a 360° rotating 10-MHz endosonographic anorectal 3D transducer (type Pro-Focus 2052; B-K Medical, Herlev, Denmark) was used to obtain axial images of the perineal muscles including the bulbospongiosus and superficial transverse perineal muscles, the external anal sphincter muscle (EAS) and the internal anal sphincter muscle (IAS). The transducer captures high-resolution transvaginal and transrectal anatomy in 60 s, and the 3D Viewer software constructs a 3D data cube, which provides measurements of distance, area, angle and volume. For 3D HRAM examinations, a rigid probe with a diameter of 10.75 mm was used (Medtronic, Shoreview, MN, USA) with 256 pressure sensors arranged in 16 rows (64 mm in length), with 16 circumferential sensors in each, and a balloon for inflation placed on the disposable sheath. Data were analysed using ManoViewAR software (Medtronic, Minneapolis, MN, USA). After anal insertion, a 1-min resting period was required before initiating measurements. Resting and maximal squeeze pressures (mmHg) were recorded for a 20-s resting period and three 5-s periods with maximum squeezing. The 3D HRAM was used as a proxy measurement for neurological damage to the pelvic floor. During the clinical examination, participants were placed in the dorsal lithotomy position without bowel preparation.

### Outcome Measurements

The primary outcome was any UI (self-reported), defined as small, moderate or large amounts of leakage, occurring twice or more times a week, no matter when the leakage occurred. This was equivalent to a score reflecting moderate or more severe UI according to recommended cut-off scores [16]. The secondary outcome was SUI 12 months postpartum according to the ICIQ-UI-SF questionnaire (questions 1, 2 and 4). SUI was defined as small, moderate, or large amounts of leakage, two or more times a week, occurring during coughing, sneezing, or physical activity/exercise. This reflected moderate or more severe SUI according to recommended cut-off scores [16].

### Perineal Tear Variables

Perineal tears were classified according to the system described by Sultan and adopted by The Green-top Guideline No. 29 [17]. First-degree tears involved injury to perineal skin and/or vaginal mucosa. Second-degree tears involved injury to the perineum, including perineal muscles but not the anal sphincter muscles. Third-degree tears involved injury to the anal sphincter complex, divided into the following categories: grade 3a tears with less than 50% of EAS thickness torn, grade 3b tears with more than 50% of EAS thickness torn, and grade 3c tears with both EAS and IAS torn. Fourth-degree tears involved injury to the anal sphincter complex (EAS and IAS) and anorectal mucosa. Labial tears were isolated to the labia.

To validate the information on perineal tears from the medical records, we used information from EVUS and EAUS on the presence of defects in the perineal muscles (yes/no) as a secondary exposure. Defects in the bulbospongiosus and superficial transverse perineal muscles were defined as any defects, whereas an EAS or IAS defect was defined as any defect greater in size than 30° of the circumference of the whole length of the EAS or IAS [18]. We also used the maximum values from 3D HRAM for each participant as a secondary exposure.

### Demographic and Obstetric Variables

Potential risk factors and confounders were chosen a priori based on directed acyclic graphs [19]. Data collected at baseline and 2 weeks postpartum, included age in years, height in centimetres, pre-gestational weight in kilograms and smoking status (yes/no) from chart reviews. The body mass index (BMI) was calculated as kg/m^2^. Pregnancy, birth and postpartum information was obtained from medical records, including gestational or pre-gestational diabetes mellitus (yes/no), duration of active labour, defined as the duration from the onset of regular uterine contractions resulting in progressive effacement and dilation of the cervix of at least 4 cm until delivery (min), duration of the second stage of labour, defined as the duration from full dilation of the cervix and onset of pushing until delivery (min), operative delivery (yes/no) and birthweight (g).

### Statistical Analyses

Baseline characteristics according to the degree of tear were presented using descriptive statistics.

Generalised linear models with binomial distribution and a log link were used to estimate relative risks (RR) with 95% confidence intervals (CI) for associations between the degree of tear, EVUS, EAUS findings and 3D HRAM findings with any UI and SUI. The same method was used to examine how age, smoking, BMI, birth weight, duration of the second stage of labour, active birth duration and operative delivery were associated with any UI and SUI. In a first model, adjustments were made for age and BMI as continuous variables, if they were not the exposure. In a second model, we used the same strategy to additionally adjust for smoking, diabetes and operative delivery as categorical variables and duration of active labour, duration of the second stage of labour, and foetal birthweight as continuous variables. The duration of the second stage and the duration of active birth were not mutually adjusted. All presented adjusted RRs (aRR) in the results section are from the second adjusted model.

All statistical tests were two-tailed, and *p* values < 0.05 were considered statistically significant. Analyses were conducted using STATA statistical software version 15.0 [20].

### Details of Ethics Approval

The study was approved by a Scientific Ethics Committee and a Data Protection Agency. All participants provided written informed consent.

## Results

### Participants

A total of 832 women were invited to participate. Of these, 81 declined and 138 could not be reached for informed consent, leaving 613 women who consented to participate in the study 2 weeks postpartum. Ten women withdrew consent, resulting in a study population of 603 women. The following three groups of women were included in the study: 203 women with no, labial or first-degree tears; 200 women with second-degree tears; and 200 women with anal sphincter tears. Women with no/labial tears, first-degree and second-degree tears were only included at OUH, whereas the inclusion of women with anal sphincter tears were expanded to all four hospitals in order to achieve the inclusion within the time limit of the study. Three women reported pre-pregnancy SUI, and five reported any pre-pregnancy UI and were excluded from the analyses.

At the 12-month follow-up, 569 of the 603 women completed the questionnaire (94.4%), with 189 having no/labia/first-degree tears, 193 having second-degree tears and 187 having third-/fourth-degree tears). EVUS and EAUS were performed on 498 women (82.6%), and 3D HRAM on 479 women (79.4%). Reasons for missing data for these two investigations were either rejection of the procedure or technical problems with the equipment.

### Characteristics According to Degree of Tear

Women with third-/fourth-degree tears were older than those with second-degree tears or no/labia/first-degree tears (Table 1). Higher tear degrees were seen with higher birthweight and head circumference, longer second stage of labour and longer active labour. The frequency of instrumental delivery was higher among women with second-degree (15%) and third- or fourth-degree tears (34%) than in those with no/labia/first-degree tears (3%). All instrumental deliveries but one was by ventouse.

**Table 1:** Characteristics according to degree of rupture among primiparous women (*N*=569)

	Total (*n* = 569), *n* (%)	No/labia/first-degree (*n* =189), *n* (%)	Second degree (*n* =193), *n* (%)	Third/fourth degree) (*n* =187), *n* (%)
Age (years)				
≤25	143 (25.1)	62 (32.8)	51 (26.4)	30 (16.0)
26–30	284 (49.9)	91 (48.2)	90 (46.6)	103 (55.1)
>30	142 (25.0)	36 (19.1)	52 (26.9)	54 (28.9)
BMI (kg/m^2^)^a^				
<25	364 (64.1)	127 (67.2)	120 (62.5)	117 (62.6)
25–29.9	130 (22.9)	40 (21.2)	46 (24.0)	44 (23.5)
≥29.9	74 (13.0)	22 (11.6)	26 (13.5)	26 (13.9)
Smoker (yes)^a^	23 (4.1)	7 (3.7)	9 (4.7)	7 (3.8)
Diabetes mellitus (yes)	20 (3.5)	5 (2.7)	8 (4.2)	7 (3.7)
Birthweight (g)				
<3,000	76 (13.4)	38 (20.1)	23 (11.9)	15 (8.0)
3,000–3,499	201 (35.3)	70 (37.0)	84 (43.5)	47 (25.1)
3,500–3,999	215 (37.8)	63 (33.3)	65 (33.7)	87 (46.5)
≥4,000	77 (13.5)	18 (9.5)	21 (10.9)	38 (20.3)
Head circumference (cm)^b^				
<34	142 (25.0)	56 (29.8)	52 (27.1)	34 (18.2)
34	121 (21.3)	46 (24.5)	37 (19.3)	38 (20.3)
35	135 (23.8)	34 (18.1)	54 (28.1)	47 (25.1)
>35	169 (29.8)	52 (27.7)	49 (25.5)	68 (36.4)
Second stage duration (min)				
<16	112 (19.7)	40 (21.2)	49 (25.4)	23 (12.3)
16–30	196 (34.5)	80 (42.3)	61 (31.6)	55 (29.4)
31–45	93 (16.3)	31 (16.4)	27 (14.0)	35 (18.7)
>45	168 (39.5)	38 (20.1)	56 (29.0)	74 (39.6)
Active birth duration (min)				
<220	141 (24.8)	68 (36.0)	47 (24.4)	26 (13.9)
221–340	144 (25.3)	44 (23.3)	54 (28.0)	46 (24.6)
341–570	149 (26.2)	50 (26.5)	50 (25.9)	49 (26.2)
>570	135 (23.7)	27 (14.3)	42 (21.8)	66 (35.3)
Operative delivery (yes)	98 (17.2)	6 (3.2)	29 (15.3)	63 (33.7)
Episiotomy (yes)	53 (14.0)	–	32 (16.6)	21 (11.2)

^a^One missing value, *n* = 568

^b^Two missing values, *n* = 567

### Risk of Urinary Incontinence

Overall, 8.6% (49 out of 568) reported any UI at the 12-month follow-up (Table 2), with 77.6% (38 out of 49) reporting moderate UI and 22.4% (11 out of 49) reporting severe UI. Another 191 women reported mild UI but were considered not to have UI in this study. Urinary incontinence was distributed as follows: 4.3% in women with no/labia/first-degree tears, 9.9% in women with second-degree tears, 9.4% in women with episiotomies and 11.8% in women with third- or fourth-degree tears. Even though more women with a tear in one or more of the perineal muscles seemed to have developed any UI than women with no/labia/first-degree tears, only estimates for women with third- or fourth-degree tears reached significance. Adjusted risk of any UI increased for women with spontaneous second-degree tears (aRR 2.04, 95% CI 0.92–4.50), for women with episiotomies (aRR 2.22, 95% CI 0.99–4.96) and for women with third-/fourth-degree tear (aRR 2.73, 95% CI 1.18–6.28), compared with women with no/labia/first-degree tears. SUI was less frequent, with 5.3% (30 out of 569) reporting SUI (Table 3), distributed as follows: 3.2% in women with no/labia/first-degree tears, 6.9% in women with second-degree tears, 9.4% in women with episiotomies and 5.4% in women with third- or fourth-degree tears. None of the differences across tear groups reached significance. Thus, the risk of SUI increased for women with spontaneous second-degree tears (aRR 2.59, 95% CI 0.65–10.3), for women with episiotomies (aRR 2.43, 95% CI 0.61–9.70) and for women with third-/fourth-degree tear (aRR 1.59, 95% CI 0.54–4.66), when compared with women with no/labia/first-degree tears.

**Table 2:** Relative risks for any urinary incontinence according to degree of rupture among primiparous women in Denmark (*N*=568)

	Any urinary incontinence (ICIQ-UI-SF)											
	Total, *n* =568), *n*	Yes, *n* =49 (8.6%), *n* (%)	No, *n* =519 (91.4%), *n* (%)	Crude			Adjusted^b^			Adjusted^c^		
				RR	95% CI	*p*	RR	95% CI	*p*	RR	95% CI	*p*
Degree of rupture												
No/labia/first	188	8 (4.3)	180 (95.7)	1.00	Reference		1.00	Reference		1.00	Reference	
Second (spontanous)	161	16 (9.9)	145 (90.1)	2.34	1.03–5.31	0.04	2.17	0.96–4.95	0.06	2.04	0.92–4.50	0.08
Second (mediolateral episiotomy)	32	3 (9.4)	29 (90.6)	2.20	0.62–7.87	0.22	2.09	0.60–7.38	0.25	2.22	0.99–4.96	0.05
Third/fourth	187	22 (11.8)	165 (88.2)	2.77	1.26–5.16	0.01	2.53	1.15–5.55	0.02	2.73	1.18–6.28	0.02
Age (years), mean (SD)	28.2	29.5 (4.1)	28.1 (4.0)									
Per 1 year older				1.07	1.01–1.14	0.02	1.07	1.01–1.14	0.02	1.08	1.01–1.14	0.02
≤25	143	7 (4.9)	136 (95.1)	0.60	0.27–1.37	0.23	0.61	0.27–1.38	0.24	0.61	0.27–1.39	0.24
26–30	283	23 (8.1)	260 (91.9)	1.00	Reference		1.00	Reference		1.00	Reference	
>30	142	19 (13.4)	123 (86.6)	1.65	0.93–2.92	0.09	1.63	0.91–2.89	0.10	1.60	0.90–2.84	0.11
BMI (kg/m^2^)^c^, mean (SD)	24.5	25.4 (5.7)	24.4 (4.5)									
Per 1 BMI higher				1.04	0.99–1.10	0.13	1.04	0.99–1.09	0.14	1.04	0.99–1.09	0.17
<25	364	28 (7.7)	336 (92.3)	1.00	Reference		1.00	Reference		1.00	Reference	
25–29.9	129	10 (7.8)	119 (92.3)	1.01	0.50–2.02	0.98	1.00	0.50–1.99	0.99	1.02	0.51–2.04	0.96
>29.9	74	11 (14.9)	63 (85.1)	1.93	1.00–3.71	0.05	1.97	1.02–3.74	0.04	1.88	0.98–3.60	0.06
Smoker (yes)^c^	23	4 (17.4)	19 (82.6)	2.10	0.83–5.35	0.12	2.35	0.94–5.87	0.07	2.65	1.04–6.79	0.04
Diabetes (yes)	19	1 (5.3)	18 (94.7)	0.60	0.09–4.13	0.61	0.51	0.07–3.53	0.50	0.48	0.07–3.33	0.46
Birthweight (g), mean (SD)	3,504	3,511 (575)	3,503 (469)									
Per 100-g gain				1.01	0.95–1.06	0.84	1.00	0.95–1.06	0.91	0.99	0.93–1.05	0.80
<2,999	76	10 (13.2)	66 (86.8)	2.03	0.93–4.44	0.08	1.87	0.86–4.09	0.12	2.20	1.00–4.86	0.05
3,000–3,499	201	13 (6.5)	188 (93.5)	1.00	Reference		1.00	Reference		1.00	Reference	
3,500–3,999	214	18 (8.4)	196 (91.6)	1.30	0.65–2.58	0.45	1.20	0.60–2.40	0.60	1.16	0.57–2.34	0.68
≥4,000	77	8 (10.4)	69 (89.6)	1.61	0.69–3.72	0.27	1.55	0.67–3.60	0.31	1.40	0.60–3.25	0.44
Second-stage duration (min),												
Mean (SD)	37	41 (28)	36 (26)									
Per 10 min longer				1.01	1.00–1.01	0.27	1.01	1.00–1.01	0.29	1.01	1.00–1.02	0.21
<16	112	13 (11.6)	99 (88.4)	2.28	1.03–5.02	0.04	2.15	0.98–4.73	0.06	2.38	1.07–5.28	0.03
16–30	196	10 (5.1)	186 (94.9)	1.00	Reference		1.00	Reference		1.00	Reference	
31–45	93	7 (7.5)	86 (92.5)	1.48	0.58–3.75	0.41	1.50	0.59–3.85	0.40	1.41	0.55–3.62	0.47
>45	167	19 (11.4)	148 (88.6)	2.23	1.07–4.66	0.03	2.33	1.13–4.84	0.02	2.50	1.16–5.40	0.02
Active birth duration (min),												
Mean (SD)	415	451 (266)	412 (265)									
Per 60 min longer				1.00	1.00–1.00	0.31	1.00	1.00 –1.00	0.52	1.00	1.00–1.00	0.41
<220	141	8 (5.7)	133 (94.3)	0.63	0.27–1.47	0.28	0.65	0.28–1.52	0.32	0.65	0.28–1.52	0.32
221–340	144	13 (9.0)	131 (91.0)	1.00	Reference		1.00	Reference		1.00	Reference	
341–570	148	13 (8.8)	135 (91.2)	0.97	0.47–2.03	0.94	1.01	0.48–2.09	0.99	1.04	0.50–2.16	0.92
>570	135	15 (11.1)	120 (88.9)	1.23	0.61–2.49	0.56	1.14	0.56–2.31	0.71	1.27	0.61–2.66	0.64
Operative delivery (yes)	98	9 (9.2)	89 (90.8)	1.08	0.54 2.15	0.83	0.98	0.49–1.97	0.85	0.85	0.40–1.83	0.68

*ICIQ-UI-SF* International Consultation on Incontinence Questionnaire-Urinary Incontinence Short Form, *BMI* body mass index, *RR* relative risk (relative risk regression), *CI* confidence interval, *SD* standard deviation

*p* values < 0.05 were considered statistically significant

^a^One missing value (*n* = 567)

^b^Adjusted for age and BMI

^c^Adjusted for age, BMI, birthweight, duration of the second stage of labour, active birth duration, operative delivery, smoking and diabetes. (Duration of the second stage of labour and active birth are not mutually adjusted for each other)

**Table 3:** Relative risks for stress urinary incontinence according to degree of rupture among primiparous women in Denmark (*n*=566)

	Stress urinary oncontinence (ICIQ-UI-SF)			Crude			Adjusted^b^			Adjusted^c^		
	Total, (*n* =566, *n*	Yes, *n* = 30 (5.3%), *n* (%)	No, *n* = 536 (94.7%), *n* (%)	RR	95% CI	*p*	RR	95% CI	*p*	RR	95% CI	*p*
Degree of rupture												
No/labia/first	188	6 (3.2)	182 (96.8)	1.00	Reference		1.00	Reference		1.00	Reference	
Second (spontanous)	160	11 (6.9)	149 (93.1)	2.15	0.82–5.69	0.12	1.91	0.72–5.03	0.19	2.59	0.65–10.3	0.18
Second (mediolateral episiotomy)	32	3 (9.4)	29 (90.6)	2.94	0.77–11.5	0.11	2.76	0.73–10.4	0.13	2.43	0.61–9.70	0.21
Third/fourth	186	10 (5.4)	176 (94.6)	1.68	0.62–4.54	0.30	1.485	0.63–3.98	0.44	1.59	0.54–4.66	0.40
Age (years), mean (SD)	28 (4)	30 (4)	28 (4)									
Per one year older				1.10	1.02–1.18	0.01	1.09	1.02–1.18	0.02	1.10	1.01–1.18	0.02
≤25	142	3 (2.1)	139 (97.9)	0.40	0.12–1.35	0.14	0.41	0.12–1.39	0.15	0.46	0.13–1.57	0.21
26–30	282	15 (5.3)	267 (94.7)	1.00	Reference		1.00	Reference		1.00	Reference	
>30	142	12 (8.5)	130 (91.5)	1.59	0.76–3.30	0.22	1.57	0.76–3.26	0.22	1.57	0.75–3.26	0.23
BMI (kg/m^2^)^a^, mean (SD)	24.5 (4.6)	26.1 (6.3)	24.4 (4.5)									
Per one BMI higher				1.07	1.00–1.14	0.05	1.06	1.00–1.13	0.05	1.07	1.01–1.14	0.02
<25	362	16 (4.4)	346 (95.6)	1.00	Reference		1.00	Reference		1.00	Reference	
25–29.9	129	7 (5.4)	122 (94.6)	1.22	0.52–2.92	0.64	1.23	0.52–2.89	0.64	1.40	0.59–3.31	0.45
>29.9	74	7 (9.5)	67 (90.5)	2.14	0.91–5.02	0.08	2.17	0.93–5.06	0.07	2.43	1.04–5.65	0.04
Smoker (yes)^a^	23	1 (4.3)	22 (95.7)	0.81	0.11–5.71	0.84	0.83	0.12–5.66	0.85	0.94	0.14–6.54	0.95
Diabetes (yes)	19	0	19	–	–	–	–	–	–	–	–	–
Birthweight (g), mean (SD)	3,503 (477)	3,430 (562)	3,508 (472)									
Per 100 g gain				0.97	0.90–1.04	0.42	0.97	0.90–1.04	0.34	0.96	0.88–1.03	0.25
<2,999	75	7 (9.3)	68 (90.7)	2.08	0.80–4.71	0.19	1.82	0.70–4.71	0.22	1.71	0.42–6.94	0.45
3,000–3,499	201	9 (4.5)	192 (95.5)	1.00	Reference		1.00	Reference		1.00	Reference	
3,500–3,999	214	9 (4.2)	205 (95.8)	0.94	0.38–2.32	0.89	0.81	0.33–2.01	0.65	1.08	0.30–3.93	0.91
≥4,000	76	5 (6.6)	71 (93.4)	1.47	0.51–4.24	0.51	1.38	0.48–3.98	0.56	2.18	0.26–17.9	0.47
Duration of the second stage of labour (min), mean (SD)	37 (27)	46 (31)	36 (26)									
Per 10 min longer				1.10	0.99–1.22	0.07	1.10	0.99–1.21	0.08	1.12	1.00–1.26	0.04
<16	111	6 (5.4)	105 (94.6)	1.32	0.47–3.72	0.60	1.22	0.44–3.40	0.71	1.10	0.39–3.14	0.86
16–30	196	8 (4.1)	188 (95.9)	1.00	Reference		1.00	Reference		1.00	Reference	
31–45	92	3 (3.3)	89 (96.7)	0.80	0.22–2.94	0.84	0.84	0.23–3.13	0.80	0.86	0.23–3.22	0.83
>45	167	13 (7.8	154 (92.2	1.91	0.81–4.49	0.14	1.87	0.79–4.42	0.15	1.98	0.81–4.83	0.13
Active birth duration (min), mean (SD)	415 (265)	431 (243)	414 (266)									
Per 60 min longer				1.01	0.94–1.09	0.70	1.00	0.93–1.08	0.99	1.01	0.93–1.10	0.77
<220	140	6 (4.3)	134 (95.7)	0.77	0.27–2.17	0.62	0.81	0.29–2.26	0.69	0.88	0.31–2.48	0.81
221–340	144	8 (5.6)	136 (94.4)	1.00	Reference		1.00	Reference		1.00	Reference	
341–570	148	7 (4.7)	141 (95.3)	0.85	0.28–2.29	0.75	0.89	0.33–2.37	0.81	0.91	0.34–2.45	0.86
>570	134	9 (6.7)	125 (93.3)	1.21	0.48–3.04	0.69	1.08	0.43–2.72	0.87	1.06	0.38–2.93	0.92
Operative delivery (yes)	98	6 (6.1)	92 (93.9)	1.19	0.50 2.84	0.69	1.06	0.45–2.55	0.89	0.82	0.31–2.20	0.70

*ICIQ-UI-SF* International Consultation on Incontinence Questionnaire-Urinary Incontinence Short Form, *BMI* body mass index, *RR* relative risk (relative risk regression), *CI* confidence interval, *SD* standard deviation

*p* values < 0.05 were considered statistically significant

^a^One missing value (*n* = 565)

^b^Adjusted for age and BMI

^c^Adjusted for age, BMI, birthweight, duration of the second stage of labour, duration of active birth, operative delivery, smoking and diabetes. (Duration of the second stage of labour and active birth are not mutually adjusted for each other)

Women with a high BMI were at increased risks of both any UI and SUI (aRR 1.88, 95% CI 0.98–3.60, and 2.43, 95% CI 1.04–5.65 respectively; Tables 2, 3). Each one-unit increase in BMI increased the risk of SUI by 7% (aRR 1.07, 95% CI 1.01–1.14), and each 1-year increase in age increased the risk by 8% for any UI (1.08, 95% CI 1.01–1.14) and by 10% for SUI (aRR 1.10, 95% CI 1.01–1.18). Smokers were at an increased risk of any UI (aRR 2.65, 95% CI 1.04–6.79). Duration of the second stage of labour was associated with UI, with a 12% increase in the risk of SUI for each 10-min longer duration (aRR 1.12, 95% CI 1.00–1.26). Compared with a duration of 16–30 min, an increase in any UI was observed with a short duration (<16 min; aRR 2.38, 95% CI 1.07–5.28) and an increase with a long duration (> 45 min; aRR 2.50, 95% CI 1.16–5.40).

Defects in the perineal muscles (internal and external anal sphincter muscles, superficial transverse perineal muscle and bulbospongiosus muscle) were all related to the development of UI, with more than a three-fold risk of both any UI and SUI in adjusted models for defects in the bulbospongiosus muscle, and more than a two-fold risk of SUI for defects in the transverse perineal muscle (Tables 4, 5). Further analyses adjusting for the degree of perineal laceration did not change the estimates.

**Table 4:** Relative risks for any urinary incontinence 12 months postpartum according to findings of endovaginal ultrasound (EVUS) with endoanal ultrasound (EAUS; *n* = 498) and high-resolution anorectal manometry (*n* = 479), among primiparous women in Denmark

	Any urinary incontinence (ICIQ-UI-SF)								
	Total, *n*	Yes, *n* (%)	No, *n* (%)	Crude		Adjusted^d^		Adjusted^e^	
				RR	95% CI	RR	95% CI	RR	95% CI
EVUS and EAUS total^a^	498	42 (8.4)	456 (91.6)						
No defect	412	26 (65.0)	386 (85.8)	1.00	Reference	1.00	Reference	1.00	Reference
Defect in BSm^b^	34	7 (18.9)	27 (6.0)	3.11	1.48–6.56	2.99	1.44–6.23	3.26	1.53–6.97
Defect in TPm^c^	39	7 (17.5)	32 (7.1)	2.44	1.16–5.17	2.48	1.17–5.24	2.42	1.14–5.16
Defect in EAS	16	0	16 (3.5)	–	–	–	–	–	–
Defect in IAS	15	1 (2.4)	14 (3.1)	0.80	0.12–5.45	0.73	0.11–4.88	0.69	0.10–4.67
HRAM total	479	40 (8.4)	439 (91.6)						
Rest pressure (mmHg), mean (SD)	77.1	64.1 (20.6)	78.3 (22.0)						
Per 10 mmHg higher, mean (SD)				0.74	0.65–0.86	0.76	0.66–0.88	0.74	0.64–0.86
Maximum squeeze pressure (mmHg), mean (SD)	155.8	137.5 (50.5)	157.5 (49.2)						
Per 10 mmHg higher, mean (SD)				0.92	0.86–0.98	0.93	0.87–0.99	0.93	0.87–0.99
Duration of squeeze (s), mean (SD)	32.5	28.6 (16.5)	32.9 (24.1)						
Per 10 s longer, mean (SD)				0.93	0.80–1.07	0.90	0.78–1.04	0.91	0.79–1.05

*ICIQ-UI-SF* International Consultation on Incontinence Questionnaire-Urinary Incontinence Short Form, *BMI* body mass index, *RR* relative risk, *CI* confidence interval, *Bsm* bulbospongiosus muscle, *TPm* transversus perineal muscle, *EAS* external anal sphincter, *IAS* internal anal sphincter, *HRAM* high-resolution anorectal manometry, *SD* standard deviation

^a^A defect in one muscle does not exclude a defect in another muscle and thus the total number of defects exceeds the number of women having undergone EVUS and EAUS examinations

^b^Ten missing values

^c^Nine missing values

^d^Adjusted for age and BMI

^e^Adjusted for age, BMI, birthweight, duration of the second stage of labour, active birth duration, operative delivery, smoking and diabetes

**Table 5:** Relative risks for stress urinary incontinence 12 months postpartum according to findings of endovaginal with endoanal ultrasound (*n* = 497) and high-resolution anorectal manometry (*n* = 479) among primiparous women in Denmark

	Stress urinary incontinence (ICIQ-UI-SF)									
	Total, n	Yes, *n* (%)	No, *n* (%)	Crude		Adjusted^d^			Adjusted^e^	
				RR	95% CI	RR	95% CI		RR	95% CI
EVUS and EAUS total^a^	497	25 (5.0)	472 (95.0)							
No defect	412	16 (61.5)	396 (83.9)	1.00	Reference	1.00	Reference		1.00	Reference
Defect in BSm^b^	34	5 (21.7)	29 (6.2)	3.71	1.47–9.37	3.41	1.36–8.58		3.85	(1.54–9.63)
Defect in TPm^c^	39	3 (12.5)	36 (7.7)	1.65	0.51–5.28	1.65	0.51–5.32		1.72	(0.53–5.55)
Defect in EAS	16	0	16 (3.4)	–	–	–	–		–	–
Defect in IAS	15	0	15 (3.2)	–	–	–	–		–	–
HRAM total	479	24 (5.0)	455 (95)							
Rest pressure (mmHg), mean (SD)	77.1	65.1 (21.1)	77.8 (22.1)							
Per 10 mmHg higher, mean (SD)				0.76	0.63–0.91	0.78	0.65–0.94		0.75	0.61–0.91
Maximum squeeze pressure (mmHg), mean (SD)	155.8	146.7 (57.1)	156.3 (49.2)							
Per 10 mmHg higher, mean (SD)				0.96	0.89–1.05	0.97	0.89–1.06		0.98	0.90–1.06
Duration of squeeze (s), mean (SD)	32.5	30.1 (17.5)	32.7 (23.9)							
Per 10 s longer, mean (SD)				0.97	0.82–1.16	0.94	0.79–1.11		0.92	0.77–1.10

*ICIQ-UI-SF* International Consultation on Incontinence Questionnaire-Urinary Incontinence Short Form, *EVUS* endovaginal ultrasound, *EAUS* endoanal ultrasound, *BMI* body mass index, *Bsm* bulbospongiosus muscle, *TPm* transversus perineal muscle, *EAS* external anal sphincter, *IAS* internal anal sphincter, *RR* relative risk, *CI* confidence interval, *HRAM* high-resolution anorectal manometry, *SD* standard deviation

^a^A defect in one muscle does not exclude a defect in another muscle and thus the total number of defects exceeds the number of women having undergone EVUS and EAUS examinations)

^b^Ten missing values

^c^Nine missing values

^d^Adjusted for age

^e^Adjusted for age, BMI, birthweight, duration of the second stage of labour, active birth duration, operative delivery, smoking and diabetes

Women with any UI or SUI had lower anal mean resting pressures (Tables 4, 5). The value was 64 mmHg in women with any UI and 65 mmHg in women with SUI, compared with 78 mmHg in women with no UI. For the mean maximum squeeze pressure, the corresponding values were 138 mmHg for any UI and 147 mmHg for SUI, compared with 157 mmHg for women with no UI. For the mean maximum duration of a squeeze, the values were 29 mmHg for any UI and 33 mmHg for SUI compared with 33 mmHg for women with no UI. For every 10 mmHg higher resting pressure, the adjusted risk of any UI and SUI decreased significantly by 26% and 25% respectively. For maximum squeeze pressure and the duration of the maximum squeeze pressure, no significant differences were found in the adjusted models.

## Discussion

### Principal Findings

This prospective cohort study adds to the knowledge of the long-term consequences of perineal tears for postpartum UI in primiparous women. Compared with women with no tears, labia tears or first-degree tears, a higher prevalence of any UI was observed in women with third- or fourth-degree perineal tears. Women with perineal tears more often reported SUI 1 year postpartum, although these differences did not reach statistical significance, probably because of the small number of cases.

### Results—in the Context of What is Known

Few studies have investigated the association between the degree of perineal tear and the risk of developing UI postpartum, making these results difficult to compare with existing knowledge. Additionally, a clear and widely accepted definition of bothersome UI does not exist, resulting in the use of heterogeneous terminology and measurement instruments [21]. However, the number of women reporting any UI following perineal tears in the present study was comparable with that found in other studies, ranging between 15% and 25% among women with third- or fourth-degree tears [22–26] and 5% to 15% among women with first- or second-degree tears [24, 27]. More women with a low anal pressure, measured by HRAM, had any UI and SUI than women with higher anal pressure. Previous studies have shown pelvic floor exercises to have a positive effect on the treatment of UI, particularly SUI [28]. As pelvic floor strength is reflected in anal pressure [29], our results are somewhat in accordance with these studies.

Our finding of a higher risk of UI in women with a high BMI is supported by several previous studies [9, 30]. The mechanism behind this association is not fully understood but is believed to be due to increased intra-abdominal pressure on the bladder and the pelvic floor, making it difficult for these women to resist the urge to urinate [31]. The association between increasing maternal age and increased risk of UI aligns with previous research, as the prevalence of UI is higher in the elderly female population than in younger women [30, 32]. Additionally, an association between UI after vaginal delivery and maternal age has been found in earlier studies [5]. The findings on the association between the duration of the second stage of labour and the risk of UI are ambiguous, with few studies available. We found that a longer duration of the second stage of labour increased the risk of both any UI and SUI, a finding supported by a systematic review and meta-analysis published in 2023 [33], although not consistently found in other studies.

Our results indicated that the increased risk of both any UI and SUI seemed to be due to defects in the superficial perineal muscles rather than defects in the anal sphincter muscles, as the risk of UI was higher among women with ultrasound-detected defects in the superficial muscles than among those with defects in the anal sphincter muscles. These findings are new, with similar results not found in other studies. However, in 2006, Borello-France et al. found that women who had an anal sphincter tear were not at an increased risk of developing UI compared with women giving birth vaginally without an anal sphincter tear or by caesarean section [12, 13]. This supports the notion that the superficial perineal muscles play a significant role in maintaining urinary continence [34].

### Strengths and Limitations

The prospective study design and inclusion of primiparous women based on the degree of tear were major strengths of this study. Women with no tears, labial tears, or first-degree tears were used as controls, allowing us to assess the association between the degree of tear and the risk of UI without the influence of previous deliveries and tears. All women had a clinical examination 2 weeks postpartum, including a perineal inspection and a digital palpation, which likely minimised misclassification according to exposure group. Additionally, the study had a high follow-up rate, with approximately 95% answering the questionnaire 12 months postpartum.

Limitations of the study include the statistical power. Although the size of each perineal tear group was relatively large, few women experienced UI, and fewer SUI 1 year postpartum. We had information on several important variables, but including them in our statistical models, where the number of exposed cases was low, may have led to overfitting, increasing the variance.

## Conclusions

This study found an association between the degree of perineal lacerations and any UI among primiparous women 12 months postpartum. Women with third- or fourth-degree tears had higher risks of any UI than women with no tears, labial tears or first-degree tears. This could be related to neurological damage to the pelvic floor or muscle damage. In this study, the increased risk of any UI seemed to be due to defects in the superficial perineal muscles rather than defects in the anal sphincter muscles. This highlights the importance of preventing and managing all perineal lacerations in the postpartum period to reduce the risk of UI.

## Data Availability

Data are available by request to the corresponding author.
